# Evaluation of the presence and zoonotic transmission of *Chlamydia suis* in a pig slaughterhouse

**DOI:** 10.1186/s12879-014-0560-x

**Published:** 2014-10-30

**Authors:** Kristien De Puysseleyr, Leentje De Puysseleyr, Hendrik Dhondt, Tom Geens, Lutgart Braeckman, Servaas A Morré, Eric Cox, Daisy Vanrompay

**Affiliations:** Department of Molecular Biotechnology, Faculty of Bioscience Engineering, University of Ghent, Coupure Links 653, Gent, B-9000 Belgium; Provikmo, Occupational Health Services, Bruges, Belgium; Department of Public Health, Faculty of Medicine and Health Sciences, Ghent University, Ghent, Belgium; Department of Medical Microbiology & Infection Control, Laboratory of Immunogenetics, VU University Medical Center, Amsterdam, The Netherlands; Department of Genetics and Cell Biology, Institute for Public Health Genomics (IPHG), Research School GROW (School for Oncology & Developmental Biology), Faculty of Health, Medicine & Life Sciences, University of Maastricht, Maastricht, The Netherlands; Department of Virology, Parasitology and Immunology, Faculty of Veterinary Medicine, University of Ghent, Ghent, Belgium

**Keywords:** Chlamydia suis, Swine, Zoonosis, Tetracycline, Public health

## Abstract

**Background:**

A significant number of studies on pig farms and wild boars worldwide, demonstrate the endemic presence of *Chlamydia suis* in pigs. However, the zoonotic potential of this pathogen, phylogenetically closely related to *Chlamydia trachomatis*, is still uninvestigated. Therefore, this study aims to examine the zoonotic transmission in a Belgian pig abattoir.

**Methods:**

Presence of *Chlamydia suis* in pigs, contact surfaces, air and employees was assessed using a *Chlamydia suis* specific real-time PCR and culture. Furthermore, *Chlamydia suis* isolates were tested for the presence of the *tet(C)* gene.

**Results:**

*Chlamydia suis* bacteria could be demonstrated in samples from pigs, the air and contact surfaces. Moreover, eye swabs of two employees were positive for *Chlamydia suis* by both PCR and culture. The *tet*(C) gene was absent in both human *Chlamydia suis* isolates and no clinical signs were reported.

**Conclusions:**

These findings suggest the need for further epidemiological and clinical research to elucidate the significance of human ocular *Chlamydia suis* infections.

**Electronic supplementary material:**

The online version of this article (doi:10.1186/s12879-014-0560-x) contains supplementary material, which is available to authorized users.

## Background

The negative influence of *Chlamydiaceae* infections on the economic yield of the pig industry is underestimated [1]. Four chlamydial species are regularly observed in the pig population: *Chlamydia (C.) pecorum*, *C. abortus*, *C. psittaci* and *C. suis*[1]. *Chlamydia suis* is the most prevalent and its primary pathogenicity is proven by several experimental infections in gnotobiotic pigs [2]-[8]. In field, this pathogen is mainly associated with respiratory disease, diarrhea, conjunctivitis, reproductive failure and subclinical infections [1],[8]. Chlamydiae are generally highly sensitive to the relatively inexpensive tetracycline antibiotics. However, the first tetracyline resistant (Tc^R^) *C. suis* strains appeared in the U.S. in 1998 [9]. Ever since, infections with *C. suis* strains have been reported on Italian [10],[11], Estonian [12], Belgian, Cypriot, German, Israeli [13], Swiss [14] and Dutch [15] pig farms. The majority of these infections were due to Tc^R^*C. suis* strains. Moreover all of these farms suffered from severe reproductive failure leading to marked economic loss.

Exposure to Tc^R^*C. suis* strains poses an additional risk for pig handlers. In fact, Suchland *et al*. [16] demonstrated horizontal transfer of the tetracycline resistance gene *tet*(C) among chlamydial species.

The use of tetracycline antibiotics as treatment for chlamydial infections in pigs, leads to selection for resistant strains [14]. Consequently, the emergence of Tc^R^ strains requires the use of other, more expensive antibiotics and, may become economically devastating to pig production. Besides the economical consequences, Tc^R^*C. suis* strains are also a potential threat to public health. Contact between Tc^R^*C. suis* and tetracycline sensitive *C. trachomatis* bacteria might lead to creation of a Tc^R^*C. trachomatis* strain in persons infected with both strains. Nevertheless, the co-infection of a person with *C. suis* and *C. trachomatis* is only possible if *C. suis* is a zoonotic bacteria. Albeit the zoonotic potential of *C. suis* is still unexamined, the zoonotic transfer of *C. suis* is a plausible hypothesis since *C. suis* is phylogenetically highly related to *C. trachomatis*, a natural pathogen of humans [17]. Therefore, the present study examines the prevalence of *C. suis* in butcher hogs (±100 kg) being slaughtered in a Belgian abattoir, and simultaneously investigates zoonotic transmission. For this purpose, slaughterhouse employees, air and possible direct contact surfaces were sampled. Pigs, air and contact surfaces were diagnosed for Tc^R^*C. suis*, while humans were examined for Tc^R^*C. suis* and *C. trachomatis.* As far as known, the present study is the first evaluation of the zoonotic potential of *C. suis* in a pig slaughterhouse.

## Methods

### Sampling of pigs and humans

Rayon-tipped aluminium-shafted swabs (Copan; Fiers, Kuurne, Belgium) were used to sample pigs and humans. For monitoring *C. suis* in pigs, rectal swabs (n=100; 10 pigs of 10 Belgian farrowing to slaughter farms) were taken upon arrival in the slaughterhouse. No information on clinical signs or medication during the fattening period was available. Sampling was performed on one day. A swab for PCR was placed in DNA/RNA stabilization buffer (Roche) and a swab for culture was immersed in chlamydia transport medium (2-SP). At the same day, employees voluntarily provided (informed consent) an ocular and pharyngeal swab, and they were asked to bring a fresh stool swab and a first void morning urine sample the next day. All samples were kept at 4°C and they were stored at −80°C upon arrival in the laboratory. Volunteers filled out a medical questionnaire, in the presence of a medical doctor, to assess information on professional (work environment of the employee) and nonprofessional activities, general health status, smoking habits, use of medication, allergies and clinical signs.

This study was approved by the medical ethical committee of Ghent University (approval EC UZG 2011/459). Participants provided their written informed consent and the consent procedure was approved by the medical ethical committee.

### Sampling of air and contact surfaces

Bioaerosol monitoring for *C. suis* was performed using the MAS-100 Eco sampler (Merck, Darmstadt, Germany) as previously described [18] at different locations in the abattoir: the pig reception area (lairage and stunning), slaughtering and bleeding area, pre-washing bath location, dehairing area, cutting/deboning area, carcass splitting area, organ evisceration area, individual weighing area, chilling/hanging room, pig intestine washing room, employee dining room, and the administrative office. In addition, also contact surfaces were sampled (water taps, door handles, tables, knives, start and stop button of machines) at all these locations by use of rayon-tipped aluminium-shafted swabs (Copan; Fiers, Kuurne, Belgium). Swabs were examined by PCR and culture. In addition, we also sampled water taps and door handles at the sanitary facilities at the individual weighing area, the pig intestine washing room and the cloakrooms.

### DNA extraction

DNA extraction on urine samples was performed by the High Pure PCR Template Preparation (HPPTP) Kit (Roche Molecular Biochemicals, Mannheim, Germany), according to the manufacturers’ protocol (version 16.0). DNA extraction of swabs and chlamydia cell culture harvest was performed as described by Wilson *et al*. [19].

### *Chlamydia suis*PCR

Pig swabs were examined by real-time PCRI, a 23S rRNA-based real-time PCR detecting *C. suis* in pigs [20]. However, this PCR analysis cannot be used for examining transfer of *C. suis* to humans, as it also detects *C. trachomatis*. Therefore, all pig and human samples were also examined by real-time PCRII, a recently developed *C. suis*-specific 23S rRNA-based real-time PCR [15]. This allowed the comparison between real-time PCRI and II, for examining *C. suis*. Similarly, all air and contact surface samples were analysed with PCRI and II. Samples with a Ct-value below 35 cycles, were retested twice. Only repeatedly positive samples were judged as positive. Genomic DNA of the *C. suis* reference strain S45 was used as positive control DNA (10^5^ particles per reaction), and DNAse –RNAse free water as negative control.

### *Chlamydia trachomatis*PCR

All ocular and pharyngeal swabs and urine samples of the employees were tested for presence of *C. trachomatis* DNA using the CE-IVD certified PRESTO Kit (Goffin Molecular Diagnostics, Houten, The Netherlands) according to manufacturer’s instructions [21].

### Culture

All samples were examined for viable chlamydial bacteria in cycloheximide-treated Vero cells using standard techniques [22]. Positive cells were enumerated in five randomly selected microscopic fields (600×, Nikon Eclipse TE2000-E, Japan) and results were scored from 0 to 6. Score 0 indicated that no *Chlamydiae* were present; Score 1 was given when a mean of 1 to 5 non-replicating elementary bodies (EB’s) plus maximum one inclusion (elementary and replicating reticulate bodies) with multiplying EB’s was observed; scores 2 to 5 were given when observing a mean of 2 to 5, 6 to 10, 11 to 15, 16 to >90% inclusion positive cells [23].

### Molecular characterization of *Chlamydia*isolates

Chlamydial isolates were molecularly characterized by real-time PCRII [15] and DNA sequence analysis of the 16S (298 bp) and 23S (627 bp) signature sequences of *Chamydia*[17]. Sequence analyses were performed by the VIB Genetic Service Facility (University of Antwerp, Antwerp, Belgium).

### *Tet*(C) PCR

*Chlamydia suis* isolates of pigs and humans were examined for presence of the tetracycline resistance gene by the *tet*(C) PCR, as described by Dugan *et al*. [24].

## Results

### *Chlamydia suis*in pigs

**Figure 1:**
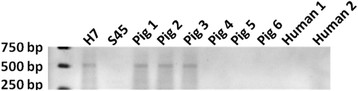
**Results of the tetracycline resistance PCR performed on swine and human** ***Chlamydia suis*** **isolates.** Three *Chlamydia suis* strains isolated from pigs were *tetC* positive. The other 12 obtained swine isolates, whereof three are represented, and both human isolates, were *tetC* negative.

Rectal swabs of 100 pigs were examined by real-time PCRI and II. Real-time PCRI revealed 45 (45%) positives. The Ct-values varied between 26.6 and 32. PCRII discovered 7 additional positives, resulting in a final number of 52 positives on 100 (52%) pigs (with the 95 percentage confidence interval ranging from 24 to 66%). This finding is consistent with the reported higher sensitivity of PCRII [15] compared to PCRI [20]. The Ct-value of real-time PCRII varied between 16.8 and 30.1. PCRII positive pigs were found on all farms. The percentage of PCRII positive pigs per farm ranged from 10 to 100%. Fifteen of 52 (28.8%) PCR positive pigs excreted *C. suis*, as demonstrated by PCRII and DNA sequencing of 16S and 23S signature sequences of obtained *Chlamydia* isolates. Those 15 culture positives originated from 8 of 10 examined farms. Three of 15 (20%) *C. suis* isolates contained the *tet*(C) gene (Figure 1). *Chlamydia suis tet*(C) positives were found on 3 of 10 farms.

### *Chlamydia suis* and *Chlamydia trachomatis*in humans

**Figure 2:**
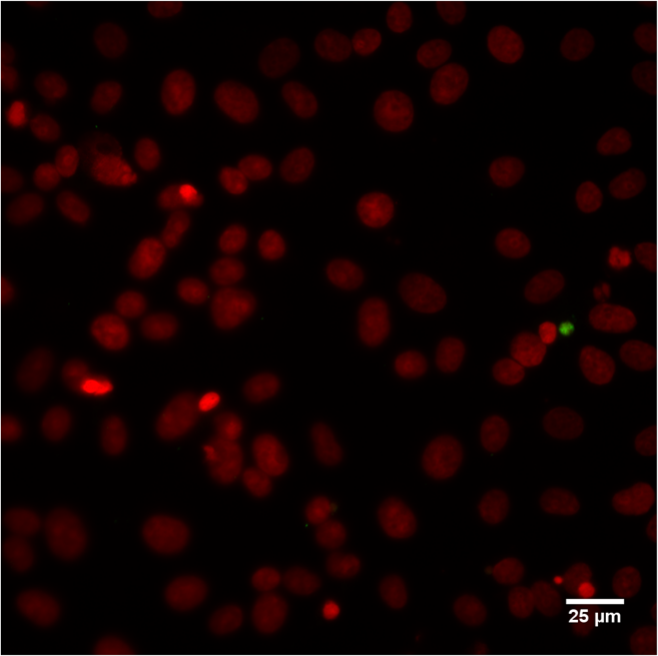
**Micrographic image of one of the obtained human** ***C. suis*** **isolates.** The larger green spot, adjacent to the red stained cell nucleus, represents a chlamydial inclusion. The small green dots are chlamydial EBs. This image corresponds to a culture score of 1. (Magnification 400×).

Only 12 of 84 (14.3%) employees participated. The age of the participants varied between 25 and 60 years with an average of 43 years. The set of 48 human samples comprised: 12 conjunctival, 12 pharyngeal, 12 stool and 12 urine samples, which were all examined for *C. suis* and *C. trachomatis.* Samples were negative for *C. trachomatis*. Pharyngeal, stool and urine samples were negative for *C. suis* by both real-time PCRI and II. However, 2 of 12 (16.6%) conjunctival swabs were positive in real-time PCRII, showing Ct-values of 26 and 28, respectively. Those two swabs were negative by real-time PCRI. Positive real-time PCRII results were confirmed by culture (both score 1), as we isolated *C. suis* from both conjunctival swabs (Figure 2). None of the human *C. suis* isolates contained the *tet*(C) gene (Figure 1). Both *C. suis* positive employees worked daily in the abattoir. One of them worked in the pig intestine washing room during the last three years while the other person did the bleeding of the pigs during the last eight years. They had no clinical signs or disease complaints while being examined by the occupational physician, nor did they mention having symptoms related to eye infections, ever since working in the slaughterhouse.

### *Chlamydia suis*on contact surfaces

Contact surfaces were all negative by real-time PCRI. Real-time PCRII could discover *C. suis* DNA, albeit small amounts (Ct-values ranging from 32.5 to 34.1) were detected on contact surfaces of nine of the 14 sampled work locations in the slaughterhouse. For six of these locations, positive real-time PCRII results were confirmed by culture (culture score 1) (Table 1).

**Table 1:** **Results of the molecular analyses on contact surface samples at different locations in the abattoir**

Location	PCRII	Viable*C. suis*
Pig reception area	**-**	**-**
Slaughtering and bleeding area	+	+
Pre-washing bath location	-	-
Dehairing area	+	+
Cutting/deboning area	-	-
Carcass splitting area	+	-
Organ evisceration area	-	-
Individual weighing area	+	+
Chilling/hanging room	-	-
Pig intestine washing room	+	-
Employee dining room	+	+
Sanitary facilities at the individual wheighing area	+	-
Sanitary facilities at the pig intestine washing room	+	+
Sanitary facilities at the cloakrooms	+	+
**Total (positives/examined)**	**9/14**	**6/14**

Swabs in DNA/RNA stabilization buffer were used to detect the presence of *C. suis* DNA by use of PCRI and II. The results of the PCRII analysis are mentioned in column `PCRII’. Swabs in 2-SP medium were inoculated on Vero-cells for evaluation of the presence of viable *Chlamydiae*. Culture positive chlamydial isolates were analysed using PCRII for subsequent molecular detection of *C. suis*. Results are shown in column `viable *C. suis*’. All culture positive samples showed isolation score 1.

### *Chlamydia suis*bioaerosol monitoring

Air samples were all negative by real-time PCRI. Real-time PCRII could discover small amounts of *C. suis* DNA (Ct-values ranging from 32.5–34.12) in the air of seven of ten sampled locations. Positive real-time PCRII results were confirmed by culture for five of these locations (Table 2).

**Table 2:** **Results of the molecular analyses on air samples at different locations in the abattoir**

Location	PCRII	Viable*C. suis*	Isolation score
Pig reception area	+	-	4
Slaughtering/bleeding area	+	-	-
Pre-washing bath location	+	+	2
Dehairing area	+	+	2
Organ evisceration area	+	+	1
Individual wheighing area	-	-	-
Chilling/hanging room	+	+	2
Pig intestine washing room	+	+	4
Dining room	-	-	-
Administration office	-	-	-
**Total (positives/examined)**	**7/10**	**5/10**	

All samples were used for direct detection of *C. suis* DNA, by use of PCRI and II (results in column `PCRII’), for inoculation on Vero cells (results in column `Isolation score’) and subsequent identification of *C. suis* (column `viable *C. suis*’).

## Discussion

The present study examines the occurrence of Tc^R^*C. suis* strains in butcher hogs being slaughtered in a Belgian abattoir and, at the same time, is focusing on zoonotic transmission of *C. suis. Chlamydia suis* infections are emerging worldwide in the pig industry [1]. Increased awareness of veterinarians and improved diagnostics might explain, albeit partially, the increasing number of reports on *C. suis* outbreaks in pigs [20],[25]. However, we are unaware if contact with *C. suis* infected pigs presents a public health risk, in particular for pig farmers and abattoir employees. After all, *Chlamydia suis* is phylogenetically highly related to the human pathogen *Chlamydia trachomatis*[17]. Moreover, both pathogens cause infections of the eye and urogenital tract in their natural hosts (reviewed by Schautteet and Vanrompay [1]). Recently, Dean *et al*. examined 101 conjunctival samples of trachoma (preventable blindness) patients who resided in a trachoma-endemic region of Nepal [26]. They found two *C. suis* infections and five mixed *C. trachomatis* plus *C. suis* infections, all leading to trachomatous inflammation. Hence, zoonotic transmission is likely.

Besides, the number of reports on Tc^R^*C. suis* infections in pigs is augmenting [9],[11],[13],[14],[27],[28]. In 2010, Schautteet *et al*. demonstrated *tet*(C) positive *C. suis* strains in 10 examined Belgian farms and in 8 on 49 (16.3%) sick pigs ending up in the autopsy room of DGZ-Animal Health Care Flanders [12]. Hence, the previously reported epidemiology of *C. suis* in Belgian pigs is confirmed by the present study since we found *tet*(C) positive *C. suis* strains in 8 of 10 examined Belgian farms. Therefore, a pig slaughterhouse is a confirmed risk environment to study the zoonotic transfer of *C. suis*. Employees provided urine and ocular, pharyngeal and fresh stool swab specimens for both PCR and culture. Two of 12 examined employees tested positive for *C. suis* by PCR and culture. However, only 12 of 84 employees participated, which was low compared to former similar studies on *C. psittaci* zoonotic transmission in poultry abattoirs [18]. Analysis of the answers on all questionnaires indicated that there were no clinical complaints, and the yearly routine medical examination revealed no clinical signs of infection, although viable *C. suis* were found in the eyes of two employees. Both individuals worked for several years in the abattoir, in the pig intestine washing room and in the slaughtering and bleeding area, respectively. Thus, exposure to blood and intestinal contents seems to present a risk for transmission to humans, but it is not strictly leading to a symptomatic course of infection. Employees are almost continuously exposed to *C. suis* and therefore could have natural immunity against disease. Serological analyses could have clarified this issue, however, employees did not give their consent for blood sampling. Contact surfaces at both locations were equally positive by both PCR and culture. Thus, *Chlamydia suis* could have ended up in the eyes through direct contact of hands with `contaminated’ contact surfaces. Besides, bioaerosol monitoring demonstrated high amounts (score 4) of viable *C. suis* in the air of the slaughtering and bleeding area. On the other hand, the air of the intestine washing room was *C. suis* negative, which could indicate that the air is not the main *C. suis* transmission route. However, further studies on larger risk populations should be conducted to get more insights into transmission routes and clinical consequences of *C. suis* in humans.

## Conclusion

The present study shows the presence of viable *C. suis* bacteria in the eyes of two employees and in air samples and contact surfaces along the slaughter line. None of the human *C. suis* isolates contained the *tet*(C) gene and both humans were negative for *C. trachomatis*. However, the adaptive ability of *C. suis* to acquire the *tet*(C) gene, especially when exposed to selective pressure, and the possibility of *C. suis* transfer to humans could have far-reaching consequences for public health. Preventive measures might reduce the risk of *C. suis* transfer to slaughterhouse employees. Besides, further epidemiological and clinical research towards human ocular *C. suis* infections is of great importance.

## Authors’ original submitted files for images

Below are the links to the authors’ original submitted files for images. Authors’ original file for figure 1 Authors’ original file for figure 2
