# Supplementary figures and images for: Evaluation of the presence and zoonotic transmission of Chlamydia suis in a pig slaughterhouse
Source: BMC Infect Dis. 2014 Oct 30;14:560. doi: 10.1186/s12879-014-0560-x (PMC4216655; doi:10.1186/s12879-014-0560-x)

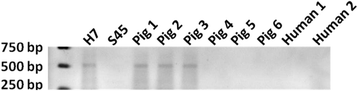

Supplement: Supplementary file 1 — Authors’ original file for figure 1 [file 12879_2014_560_MOESM1_ESM.gif]

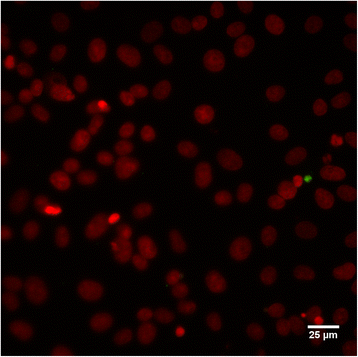

Supplement: Supplementary file 2 — Authors’ original file for figure 2 [file 12879_2014_560_MOESM2_ESM.gif]
